# Medical tourism in india: perceptions of physicians in tertiary care hospitals

**DOI:** 10.1186/1747-5341-8-20

**Published:** 2013-12-17

**Authors:** Imrana Qadeer, Sunita Reddy

**Affiliations:** 1 Center of Social Medicine and Community Health, School of Social Sciences, Jawaharlal Nehru University, New Delhi 110067, India

## Abstract

Senior physicians of modern medicine in India play a key role in shaping policies and public opinion and institutional management. This paper explores their perceptions of medical tourism (MT) within India which is a complex process involving international demands and policy shifts from service to commercialisation of health care for trade, gross domestic profit, and foreign exchange. Through interviews of 91 physicians in tertiary care hospitals in three cities of India, this paper explores four areas of concern: their understanding of MT, their views of the hospitals they work in, perceptions of the value and place of MT in their hospital and their views on the implications of MT for medical care in the country. An overwhelming majority (90%) of physicians in the private tertiary sector and 74.3 percent in the public tertiary sector see huge scope for MT in the private tertiary sector in India. The private tertiary sector physicians were concerned about their patients alone and felt that health of the poor was the responsibility of the state. The public tertiary sector physicians’ however, were sensitive to the problems of the common man and felt responsible. Even though the glamour of hi-tech associated with MT dazzled them, only 35.8 percent wanted MT in their hospitals and a total of 56 percent of them said MT cannot be a public sector priority. 10 percent in the private sector expressed reservations towards MT while the rest demanded state subsidies for MT. The disconnect between their concern for the common man and professionals views on MT was due to the lack of appreciation of the continuum between commercialisation, the denial of resources to public hospitals and shift of subsidies to the private sector. The paper highlights the differences and similarities in the perceptions and context of the two sets of physicians, presents evidence, that questions the support for MT and finally analyzes some key implications of MT on Indian health services, ethical issues emerging out of that and the need for understanding the linkages between public and private sectors for a more effective intervention for an equitable medical care policy.

## Biographic sketch

### Imrana qadeer

Imrana Qadeer’s areas of research interests are organization of health services, Primary Health Care, Political Economy of Health, Epidemiology, Health Policy Analysis, Research Methods, Systems Research, Women’s Health and Nutrition. She has published her research in national and international journals and books. Her forthcoming book is Health of the Dalit Women in India.

As a Professor at the Centre of Social medicine and Community Health, School of Social Science, Jawaharlal Nehru University, New Delhi she taught and guided research in public health for 34 years. Over 2008-2010, she was offered the prestigious J P Naik Senior fellowship by the Centre for Women's Development Studies, New Delhi. An MD in Pediatrics and a clinical registrar at the All India Institute of Medical Science, New Delhi she moved into public health to develop a critique of the bio-medical approach to public health in India. She worked with several formal institutions such as, the Planning Commission, the Population commission, and the monitoring Committee of the National Rural Health Mission. She was a member of the Standing Committee, currently a visiting Prof. at Council for Social Development of the University Grants Commission on Women’s Studies Centres in Indian Universities and is also associated with several grass root organizations working on health issues.

### Sunita Reddy

Dr. Sunita Reddy is an Anthropologist, specialized in medical anthropology, currently teaching in the ‘Center of Social Medicine and Community Health’, School of Social Sciences, Jawaharlal Nehru University. Dr. Reddy has been teaching and researching on Public Health issues. Her areas of research are women and children’s health, public private partnership in health services, Disaster studies and Medical Tourism. She has done a longitudinal research on Rehabilitation Post Tsunami in Andaman and Nicobar Islands and published a book ‘Clash of Waves’ by Indos Publishers, New Delhi (2013). She has published research papers widely in peer reviewed journals and has presented papers in many national and International conferences. She has been delivering lectures for Academic Staff colleges, for Indian Foreign Service probationers and other institutions. She is a Core group member, in formulation of guidelines on psycho-social health, widows and children in disasters, for National Disaster Management Authority (NDMA) and also member in expert group for “CBRN Disaster Management: A Step Towards Capacity Building & Resilience in Industries” throughout the country.

## Introduction

Medical Tourism in Asia is estimated as worth $ 4 billion by the year 2012 [1]. The critical factors that led to cross border travel to seek superior medical care are the rising cost of care in the developed world and presence of uninsured or underinsured people there. Patients from developed world are now seeking care in countries where hi-tech is available and inexpensive. Huge cost differentials (Asian countries charging 20% of the cost of US and UK), cheaper airfare, ‘world class’ hi-tech medical care today offers business opportunities for corporate hospitals in Asian countries.

Medical Tourism (MT) in India can be traced to the increasing numbers of corporate hospitals in the metropolitan cities. With increasing liberalization since the 80s, tertiary care in India’s health sector was opened to private sector provisioning and public institutions permitted to accept private investments in the hope of enhancing revenues [2]. The Tenth and Eleventh Plans [2,3] welcomed MT, commercialisation of the public sector and a hi-tech tertiary sector market as a part of reforms. The drafts of the National Health Bill [4] and the 12^th^ Five year Plan of India emphasised the role of private sector in medical care [5]. This privatization and commercialization that transformed medical care from a service to a commodity was a conscious policy decision accompanied by a range of subsidies including land, equipment and drug imports etc. [2,3,6]. It also attracted medical experts from the public hospitals who had received state supported medical education [7]. Like any other business in welfare services in India, medical industry also started attracting clients through capturing the high-tech services. The health services were transformed from a national medical priority based network of institutions to one that offered the services- elective (could be planned in advance) and tuned to the needs of the global patients.

Thus, the emergence of medical tourism within India’s policy frame at the turn of the century cannot be seen as an outcome of international influences alone. The ideological underpinnings of the Indian State itself are reflected in the policy shifts towards commercialisation and growth of MT that transforms health services into a source of trade, gross domestic profit, and foreign xchange [8]. The government policy of merging medical expansion and tourism was announced by Finance minister in his 2003 annual budget speech, where he called for ‘India becoming a global health destination’ [9]. Thus chains of such institutions and medi-cities got underway. India’s XIth Plan mentions a list of corporate hospitals, which provide high-end health care services through business process outsourcing [3], p.275 and the XII Plan depends heavily on the public private partnerships in financing of tertiary care as a way to handle the financial challenge [5]. Confederation of Indian Industry (CII) and Indian Health Care Federation (IHCF) wants to establish an Indian health care brand synonymous with ‘safety, trust, and excellence’ [10]. The good public sector tertiary hospitals too are being geared to practice MT. The assumption behind these policies is that the revenue thus, generated will add to the economic growth that is the basis of welfare. The fast growth of private tertiary sector hospitals and their use indicates profit maximisation for medical business and need satisfaction for the upper middle classes, but what of the physicians among them trained in the science of medicine and public health? Are they satisfied and aware of emerging ethical contradictions? These are issues this paper helps us answer.

The paper focuses on the perceptions of MT among senior physicians around four areas of concern: their understanding of MT, their perceptions and views on the hospitals they work in, the value and place of MT in their hospitals and the implications of MT for medical care in the country. The validity of their perceptions is then examined against available literature on the subject. By contrasting these views of public and corporate tertiary care physicians the paper also highlights their contrasting ethics of medical care.

## Methods

This study focuses on the perceptions of MT among senior physicians from selected public and corporate tertiary hospitals. Though based on a relatively small sample of 91 physicians, it is critical as these physicians influence the health policies through their influence on public opinion specially the middle class, their advisory and consultancy roles in health planning at the Ministry of Health and Family Welfare of the Centre and State Governments, and micromanagement of their departments and institutions. The study is based on in-depth interviews as its main tool along with observations, hospital records and secondary literature. Open-ended interview schedules shown in Additional file 1 and probes in Additional file 2, wherever necessary were used. The interviews on an average took one hour and the responses were noted verbatim.

Two reputed public and two well known corporate hospitals in each of these three cities were selected purposively. Till 2010, there was no Ethics Review Board constituted for social science research either at the School of Social Sciences or at the Jawaharlal Nehru University Level. Following the general ethical guidelines practiced in academics the authors took written consent from the hospital authorities and verbal consent from the physicians to interview and publish their views. Anonymity of both- the physicians and the hospitals is maintained. Physicians from four public and six corporate tertiary hospitals from three metropolitan cities - Delhi, Chennai and Hyderabad - were selected. In Chennai, public hospitals refused permission due to some problems with the media just before the fieldwork in 2006-2007. The departments of cardiology, orthopaedics, surgery, and paediatrics with high focus on MT, other departments with or without MT and medical administrators were also chosen for interviews. The selection of physicians was thus purposive depending on their willingness for the interview. While writing, the method of paraphrasing multiple quotes was used both for bringing out commonalities and differences between the physicians of public and corporate tertiary sectors.

Table 1 gives the distribution of physicians in the study sample. The interviews from the corporate hospitals in three cities covered 52 physicians and from public sector hospitals in two cities covered 39 physicians.

**Table 1:** Distribution of physicians interviewed across type of tertiary care hospitals in three cities

**Cities**	**Delhi**		**Hyderabad**		**Chennai**	
Physicians	Public	Corporate	Public	Corporate	Public	Corporate
MT Centred Specialities*	16	12	7	6	-	19
Specialities with or without MT**	9	1	2	2	-	7
Administration	3	2	2	3	-	
Total	28	15	11	11	-	26

*Specialities from Cardiology, Orthopaedics, Surgery and Paediatrics.

**Specialists from General Medicine, urology, neurology, gastroenterology, oncology Geriatrics and Preventive and Social Medicine.

## Findings

### Understanding of medical tourism

Physicians in the public and corporate sectors viewed MT as ‘*attracting business through marketing*’. It was ‘*travel from home to another country for treatment where the host country derives financial benefit from their travels*’. The difference was in their attitude towards competition and benefits.

The corporate sector physicians emphasised three main reasons for the popularity of MT: ‘*providing opportunities to overseas patients to avail the hi*-*tech medical facilities in India at a low cost*, *through skilled physicians who can compete with physicians in the USA and the UK*’; ‘*quality services are cheaper in India*’ with ‘*culturally similar conditions for neighbouring countries*’; and ‘*due to post*-*11 September visa restrictions to the USA*, *the Arabs and Africans prefer India*’. Some also opined that ‘*aspects like arrival*, *pick up*, *transport*, *and reception at airports ensure patient satisfaction and help standardise price structure*’. Others went to the extent of proclaiming: ‘*We deliver caring services*’; ‘*India is known for its hospitality and there are no racial differences here*’; ‘*Indians are by nature compassionate and brainy*, *so we can tap this potential*’; ‘*We are proud of our medical expertise*’; and ‘*We have state of the art equipment comparable with the best in the world*’.

Public sector physicians were more detached with a “not-for-us” attitude with lesser involvement in the competition around MT. While they all felt, ‘O*verseas patients come to public hospital for simpler treatment due to lack of good quality tertiary care in their own country or long queues*’, only those in one Delhi hospital with greater exposure to foreign clients felt, ‘*Medical tourism is a new term for an old phenomenon as our hospital has been treating foreign patients for long*’; ‘*The volume of overseas patients is not high and they pay like the private patient*’. Use of the term tourism with clinical work made these physicians uncomfortable. ‘*Tourism is always pleasure related*, *whereas MT is not*’; ‘*only traditional healing centres could be identified for promoting health tourism*’. Thus, the majority underplayed the commercial underpinning of the word ‘tourism’, where the notion of profit from service was critical as they saw them as reinvestments!

### Professional perceptions on work load and conditions in tertiary public and corporate care hospitals

Long queues for OPDs in public hospitals without a place even to stand, beds occupied by more than one patient in three out of four hospitals, stood in contrast to the decorated spaces and low patient load of the corporate hospitals. Public hospital OPDs had 1,500- 8,000 patients every day as compared to 100 to 1,200 per day in corporate hospitals. The bed strength of the former ranged between 1,500- 2,500, while the beds in corporate hospitals varied from 130 to 300.

In public hospitals, inadequacy of funds, political interference in recruitment and promotion leading to mistrust, and undermining the autonomy of institutions were a constant refrain. The internal problems reported were: unhealthy work culture, inefficiency of staff, trade unions without a strong sense of responsibility. A self-critical reputed senior doctor said, ‘*Physicians lack spirit of academic pursuits*, *transparency and autonomy in research in a disturbed work culture*, *therefore*, *quality of care gets affected and outputs are often irrelevant to our health needs. There is also a disjunction between what we do and what our national priorities are*’. Others agreed that these problems affected medical care for the poor negatively. ‘*Patients from other states*, *especially from rural areas*, *live on roads or Dharamshalas* (*inns*), *paying Rs20*-*Rs100 per day. CGHS patients wait for surgical treatment despite doctor*’*s orders for admission*, *for lack of beds. The wait is on an average 6 months* - *sometimes patients die. There is paucity of blood and Social Welfare Fund schemes are often misused by administration and politicians as few people know of these*’.

Dissatisfaction was expressed by public sector physicians in many ways, ‘*We don*’*t have adequate medical facilities for the poor in India*; *without subsidy or insurance 70*% *of the population cannot afford super specialty treatment*. ‘*Physicians are mostly overburdened*’, ‘*We have to first meet proper standards of bed and nurse availability*’. ‘*The working conditions are so poor that*, *at the end of the day*, *it is the love for one*’*s work which sustains one in such an environment and individuals focus only on what they can do*, *forgetting the macro picture*’. ‘*Public sector has reached a point where a deliberate policy to improve it is unavoidable if standards have to rise*’. A self critical perspective was, ‘*the patient has no rights*, *at times we are arrogant*, *so do not seek active participation from the patient in choosing from the options available to him*’.

In contrast, the corporate sector physicians were satisfied with their working conditions. They emphasised the need to work in a technically equipped work place where they get job satisfaction and are able to practice scientific medicine. They pointed out that their institutions offered differential packages for different income groups. Another advantage they saw was in using their skill to the maximum by taking up consultancies in more than one hospital.

### Public sector doctor’s perception of Mt

Out of the 39 interviews of public sector physicians, three sets of opinions emerged on the promotion of MT in public hospitals discussed below. The majority, i.e. 29, were very positive about the scope of MT in India. 14 of these (35.8%) thought it should be promoted even in the public sector while 15 (38.4%) felt public sector was in no position to compete and only the private sector could take advantage of it. Only eight physicians (20.5%) were critical of MT as a strategy. Two (5.1%) did not comment.

### Against MT in principle

The eight physicians against MT in principle, considered MT as, ‘*a crime against the ordinary Indians*, *an undemocratic policy*’, ‘*practiced without any consensus or meeting of all parties*’. ‘*There is a need to look at our own health needs against which the idea of MT in public hospitals* – *that were not meant for making profits* – *makes no sense*.’ ‘*Have we treated all Indians that we want to invite patients from abroad*?’ ‘*The poor do not get any benefits from the Foreign Exchange*.’ ‘*The promotion of MT will not improve the country*’*s health when we are not able to provide basic services to the people*’. They added, ‘*Our hospital focuses on education*, *research*, *and services and will resist money making policies like MT*’. They believed that, ‘*Medicine is for services and not for business. MT is against the oath of Hippocrates and therefore unethical*’; ‘*it undermines the* ‘*very basis of faith between physicians and patients*’, ‘*our institution has the capacity and quality to promote MT*, *but the institution is not meant for private service*… *If we spend so much time and energy on few patients it will deviate from the norm*/*protocol of the institution*’. Proud of the public sector’s ability to provide hi-tech 24 hour care without profit, they said, ‘*Nobody has the time to provide ancillary services*, *when key services have to be ensured*!’ Facilities like deluxe rooms and personal security, required by medical tourists, were inconceivable for them.

‘*Resource mobilization by the State*’, ‘*political will*, *appropriate priorities*, *regionalisation and internal tourism*’ was their answer to inequity, reflecting an acceptance of some degree of the commercialisation which they criticised otherwise. Despite their frustration with state policies, and their view that, ‘*MT takes advantage of lack of proper regulatory authority or effective laws and implementing agencies and makes malpractice possible*, *both in the use of technology and pricing*’, they barely contested the State support to MT in the private sector, or pointed towards its international business connections.

They wanted cross border medical treatment for humanitarian reasons and to promote international relations and not MT. For them the issue was not competing for patients in the medical market, but their inability to fully provide for their own patients and the patients’ inability to afford payments. ‘*The Ministry and the government want us to generate our own revenue but we are not getting into the market*’. This moralistic admonition of commercialisation was reflected by a doctor’s outburst, ‘*Our ethics are bad*, *we let Indian physicians go abroad and invite patients from abroad when we ourselves have surplus patients*’.

Thus 35.8 percent of the public hospital physicians felt they could use MT to the benefit of patients and professional while 38.4 percent felt that in the present form public hospitals were ill suited for MT and would further deprive the general patients, 20.5 percent were in principle against MT in public hospitals.

### MT in corporate hospitals alone

Fifteen public hospital physicians considered MT a legitimate function of the corporate hospitals (with their ‘spare capacity’). Public hospitals were seen as ‘*ill equipped*’, ‘*already over burdened*’, and ‘*ill suited*’ for it. The objections were two. Firstly: ‘*MT is going to create a divide by diverting resources from the poor who will suffer more*, *it will enhance the burden of the poor patients unless the government separates the two sets of institutions*’; ‘*The onus of care for the poor lies with the public sector and it should look after the interests of the general public*’. Secondly, they argued that: ‘*Given the present level of investments in public hospitals it is out of the question to achieve the standards needed for MT*’; ‘*The importance given to medical tourism will certainly affect general health care and public hospital is for all and not for some specific people*’. A doctor said, most physicians expressed their anguish at the state of Public health services, and emphasised the inadequacies, which need to be catered to before embarking on MT. They were clear that, ‘*MT can be encouraged and promoted in corporate and private tertiary hospitals and it would help the economy and the growth of hi*-*tech in India*’.

### MT both in public and corporate institutions

Nine specialists argued that India has the best low cost medical expertise in South Asia for treating the overseas patients and generating high revenues. They saw a tremendous scope for MT especially in plastic surgery, orthopaedics, and other non-invasive techniques. ‘*Wherever these departments are well established and of international standards*, *they can be opened for MT*.’ In Hyderabad, one of the public hospitals was planning to open a 7^th^ floor for foreign patients to promote MT - especially for NRIs. The remaining five specialists felt that primary and secondary health care should be under the public sector, while tertiary care should be through private sector and the pay clinics of government medical colleges and teaching institutions which should provide subsidised/free care for the poor.

The inter-state movement of patients from less developed states and towns to the metropolitan cities was viewed as yet another possibility for internal MT and an opportunity to improve hospital infrastructure, services, and research facilities within the public sector tertiary institutions. Some also argued that, ‘*A government doctor works from 9 a.m. to 4 p.m. and rest of the time many physicians do not have anything to do but are not allowed to practice privately. Why does the government not utilise the idle hours of the physicians who want to work extra time by opening* ‘*pay clinics*’ *in the evenings*? *Pay clinics can be operated in the same hospital by developing good infrastructure. In this way*, *the government will get substantial amount of revenue. This can be utilised for development of existing departments. Otherwise*, *the government is losing a good hand*’. The contention was that, ‘*In the government hospitals*, *there is a need to have more incentives*’. Others disagreed and argued that, ‘*Physicians in public hospitals work approximately 12 hours a day and to promote MT we need to invest in new infrastructure and manpower as the same set of people cannot meet the additional load*’. This was critical so that, ‘*we do not bleed existing resources*, *both human and mechanical*’. It was also claimed that, ‘*This would not be because of any lack of professionalism in the public sector but due to their poor infrastructure and support systems. The public hospital environment is too dirty to attract foreigners*, *in fact*, *it prevents people from coming*’.

They also wanted to have hi-tech facilities like telemedicine which they considered necessary - ‘*if patients have to be managed at a distance*’, ‘*be able to compete with corporate sector physicians*’, and ‘*see different types of patients*’. Further, ‘*quality of care may improve because of the need to meet western standards*’. This set of experts wanted to simplify laws of organ transplant and make donors easily accessible. They felt that people come to public sector hospitals because of the doctor’s name; for MT, the entire infrastructure has to improve along with ‘*quality of care*, *values and ethics*’.

They argued that the public hospitals too can generate revenue through MT by overcoming some infrastructural deficiencies. Holistic healing and preventive care should be given priority under MT and they suggested package deals of ‘*services with free accommodation*’ to Medical Tourists. The notion of ‘*generating money*’ for the public hospitals through MT was generally accepted.

Interestingly, those who supported adoption of MT and welcomed markets in public sector medical care did accept that the public sector hospital is the only recourse for the poor. They argued that improving infrastructure will help the poor as well, since the profits would be ploughed back into their care if it could be ‘*ensured that no discrimination happens between patients*’. The main arguments for promotion of MT in public hospitals were that: a) it would lead to healthy competition and inter-state tourism; b) equipment and infrastructure would be updated and new technology could be brought in for all; c) a learning opportunity for physicians; d) improved quality of care for all; f) revenue generation for the government; g) arrest brain drain from public hospitals; h) a cure for poor administration and inadequate working conditions.

### Perception of physicians in corporate hospitals

90.4% (47) physicians of the corporate hospitals saw a huge potential for MT. None talked about the public sector, which they said, ‘*is the responsibility of the State and is not our concern*’. A minority of 9.6% i.e. five physicians were critical of MT and were upset with its promotion, they reflected, ‘g*iven the poor medical facilities in many parts of the country*, *the first important thing to do is to indulge in some soul searching before inviting people from abroad*’; ‘w*e need to serve our own people*’, or that, ‘*MT is like selling India in its naked poverty*’. One specialist said, ‘*International insurance companies will really benefit from the idea by saving costs and time of the insurance cover*, *they will look for cheaper options*’. Two of these experts, in a missionary tertiary care hospital, added, ‘*we are trying for ISC* (*International Standards Certification*) *to promote MT*… *for private hospitals what matters is money*!’ Their views were however overshadowed by the MT enthusiasts who felt, ‘*An excellent concept that can put India at the top by focusing on specialty treatment*’. The range of concerns expressed by them was the following:

### Finding clients

‘*If Thailand can attract 2 million medical tourists per year*, *India should attract 20 million*’, said the Chairman of a leading corporate hospital. Another vision was to see India as, ‘*the health care hub of the east*’. The logic, ‘*India has human capital*, *is human friendly*, *and has an infrastructure which can be expanded. India needs to progressively exploit this potential*’. Another opined, ‘*MT has picked up in Mumbai*, *and Delhi is not far behind*’, and that, ‘*it would be beneficial for the country*’. An invitee to a consultation on Britain’s National Health Service by the NHS, UK, he had recommended that NHS should send patients to India for cheaper treatment. He said, ‘*It will be the underprivileged population in UK that would be sent to India for medical treatment. It*’*s a win*-*win situation forprivate hospitals to attract patients*, *earn money*, *be competitive*, *enhance their standards of care*, *and generate employment*’. In the process the bigger medical corporations might wipe out the smaller less competitive institutions he added. However, estimating the vast potential of MT, another doctor opined ‘*In Britain alone*, *there are 10 million patients who are in the waiting list in NHS*, *out of which only 11*% *are British and rest are ethnic people with 80*% *having private insurance. This is the group which we need to cover* - *ethnic and privately insured*’. Others proposed targeting the uninsured overseas clients and holding health festivals in client countries like Oman and Maldives.

### Benefits of MT

These experts saw innumerable benefits of promoting MT, specially, in areas such as dentistry, cardiology, orthopaedics, cosmetic surgery, and paediatrics. The most obvious benefit was, ‘*earning foreign exchange*’ or ‘*more the patients*, *more the income*, *which will eventually lead to growth of business*.’ Many felt, ‘*With up*- *gradation of facilities*, *health service standards will go up automatically*’. MT was also ‘*an opportunity to treat different sets of patients having different clinical problems to acquire knowledge and hone our skills*’. It was ‘*a boon to learn new techniques and handle more complicated and as yet incurable diseases*’, with the incoming technologies. They argued that,’ *60*% *of employment is generated through service sector all over the world*, *so*, *by making our institutions globally competitive*, *we can generate employment and retain competent physicians*’. MT, they said, would also create a ‘*friendly international atmosphere*’: its commercial value required that its growth be ‘*exponential*’!

Other ways suggested to promote MT were: by standardizing and getting accreditation, direct flights to the cities, improving roads, easy money transfers, medical visas and systematic and planned scaling by the state as is being practiced already.

### Physicians view of implications of Mt for medical care in India

Most experts in corporate institutions saw their own sector as the most critical in medical care and earnestly believed that, ‘*by selling Indian health industry abroad*, *and by marketing health care*, *one can increase the earnings and utilise that money in improving the existing infrastructure*’. ‘*This would force the inefficient public sector to compete and thereby raise its standards and improve job opportunities*’. This set obviously had no consideration about where the public sector resources and their own subsidies came from. Only a minority of five accepted that, ‘*by getting patients from abroad*, *dollars will get priority and the less privileged Indian patients will take a backseat*’ but they did not talk of implications for public sector.

Among those for MT, the perceived implications were positive as discussed earlier. Some even argued, ‘*MT may actually be stopping the brain drain of physicians going to work abroad by opening avenues in private sector*’. Others felt, ‘*Infrastructural improvement will improve services for all*’.

Experts against introduction of MT in public sector pointed out the negative implications of MT, such as: reduced medical care for the poor due to diversion of manpower to the care of overseas patients within the institution, as well as ‘*probable rise in malpractice by corporate hospitals by not fulfilling the condition of 25*% *free admissions to patients and thereby increasing the load on public hospitals*’. Thirdly, in MT, ‘*the preventive component of medicine is left behind*’.

Yet another concern was brain drain out of the public hospitals. Even though it was said, ‘*We cannot stop them as there is a difference in salaries*’ *or*, ‘*As such there is no mass exodus*’, Others reaffirmed ‘*an internal brain drain*’; ‘*a lot of our expert staff*, *physicians and paramedics leave public hospitals for the private sector*’. ‘*Most of the physicians working in XXXX*,^*a *^*XXXX are from government hospitals*’. ‘*Our hospital gets some of the best junior residents from all over the country*, *and it is used like a nursery for training before they join the private sector*’. Other than money, the reason for leaving the public sector was said to be ‘*eagerness to learn more and use sophisticated technology like robotic surgery and not getting stagnated*’. These views matched the finding of the study as more than fifty percent of physicians working in corporate hospitals had earlier served in public hospitals. In Chennai, 13 out of 23 physicians (3 did not respond); in Hyderabad, 5 out of 11; and in Delhi, 10 out of 15 had served in public hospitals.

Among opponents of MT there were some who did not think it would have any impact as their institution was capable of fairly handling its patients. Others said, ‘*If it is only in private hospitals then*, *that being a separate sector*, *there is no implication for general patients*’. According to them, promotion of MT in private sector may, in fact, help the poor as the growth of private sector diverts the middle and upper middle class patients away from public hospitals, which can then exclusively cater to the poor. ‘*Rather than the poor*, *the rich will be affected by MT*, *as many times*, *preference is given to foreign patients who have higher paying capacity rather than rich Indian patients*’.

These views were no doubt contradictory among the physicians for and against MT across the two sectors. Even among the opponents of MT in the public sector, the articulation of the significance of shifts of state subsidies and policy of commercialisation was muted. They saw their challenge as one of ethical practice, getting State resources and problems of micro-management.

## Discussion

Historically, the middle class – especially physicians and bureaucrats, tend to play a key role in the evolution of policy for medical care [11]. Our study reveals certain trends in the perceptions of selected senior medical physicians that are critical: as pointed out earlier this group of physicians contributes to the formation of public opinion, national policy and programs and shapes institutional traditions and organisation.

### Doctor’s Perceptions: differences and similarities & ethical underpinnings

An overwhelming majority (90%) of physicians in the private sector held the State responsible for the care of the larger population. In contrast, the public sector physicians’ daily challenge of saving lives within the constraints of their patients’ economic status and the meagre facilities available, made them sensitive to the problems of the common man. The glamour of hi-tech associated with MT however dazzled most physicians of both sectors. All physicians of the public hospitals under study felt responsible for the poor, 59 percent (23) acknowledged the irrelevance of MT for common patients and potential problems with MT in public tertiary hospitals, while 36 percent (14) thought it would improve overall patient care due to added revenues 5.1 percent (2) remained non-commital.

The economic and political linkages and dynamics between public and private sectors were largely missing from the doctor’s perceptions which was primarily limited to micro management of institutions. The disconnect between their concern for the common man and their views on MT was due to the lack of appreciation of the continuum between commercialisation and MT, and the link between denial of resources to public hospitals and shift of subsidies to the private sector [12]. The profit motive behind MT was recognised, hence, those who did not favour MT did it mostly on moral and ethical grounds.

Interestingly, only the private sector physicians, and those public sector physicians who supported MT in their institutions, talked frankly about the existing and possible links with the international market and its potentials for earning revenues, the need to consolidate the medical industry and of State subsidies to push MT without acknowledging the negative impact of the shift of state subsidies and of the logic of market forces which undermines epidemiological priorities. In contrast those in the public sector opposing MT in principle, underlined the importance of policy changes to mobilise resources for the public sector and rebuild the culture of service.

Private sector physicians were very articulate about their views on the role of the State. They demanded State support to promote MT and believed that the “competition” thus offered would force the public sector to improve! This contorted logic completely ignores the fact that the public sector was set up with State subsidies to be able to run services that are non-competitive and based on epidemiological needs, and that costs were standardised by the State to achieve equity in access. Without State subsidies it would collapse, as commercial competition shifts the emphasis to services like Obstetrics, Orthopaedics, Cardio-thoracic surgery transplants, and Urology etc. and their required manpower that distort priorities within public hospitals and health system as such [8,13,14]. The public sector experts, though wary of perpetual shortages, accepted the State’s indifference and had little to say about its constraints though, a few of them talked of reforming the medical care system.

Unaware of the massive indirect shifts of resources that are being proposed [12], the physicians in this study saw public and private sectors as discrete entities. Almost all (94.7%) opposing MT in public institutions granted corporate sector, the right to use MT to generate revenues as they believed, ‘*It adds to India*’*s economic growth*’. They did not ask how inclusive is this strategy for economic growth except for one who believed, *MT operates outside the public system*, *the Foreign Exchange earned stays in the private sector*, *and the country*’*s poor do not ever benefit*’. The others did not raise the issue of declining resources due to state support to private sector. These views illustrate that the physicians were not familiar with the shifting overall pattern of state financing of health services and it’s links with private sector, so well known to analysts of the health service system [7,15]. Most of them in one voice spoke of the huge potential of MT in India as a vehicle for economic growth while only 10 percent in the private and 20.5 percent in the public sector expressed discomfort with the very strategy of MT in their institutions.

Seen in the framework of domains of micro ethics (relationship with, care, autonomy and choice of patient) and macro ethics (issues of equity, justice, resource allocation, care of the vulnerable and maximum benefit for largest number) provided by Nancy M. Baum [16] and Calman and Downie [17], our findings indicate that private tertiary sector doctor’s concerns are largely limited to the micro ethical domain related to standards of care and management, regulations, doctor patient relations, beneficence etc. Only 10 percent of them referred to some macro ethical issues of MT as part of public health-such as needs of the majority and concern for the poor. It was primarily the public sector physicians who at all mentioned macro-ethical issues covering financial constraints, equity for the most vulnerable sections, priorities in technological choices for provisioning of services in public institutions of tertiary care and need for primary health care. Even among them 38.4 percent who wanted the state of the art technology and MT to mobilise revenues for the general patients- like 90 percent of their counterparts in the private sector had no problem with medical care becoming commodity and a part of the market. This however was under the impression that MT can mobilise resources for general patients. Thus, they saw no conflict in macro ethical issues and MT.

### Implications for state policy

A weak professional resistance to MT among senior physicians gives the state a better chance to push forward its neoliberal policies and MT, a logical outcome of the market principle, now extends the boundaries of tertiary health care market. The projected Rs.8,50,000 crores contribution to the GDP by 2020, if the Government supported it, prompted the Union budget for 2003-2004 to give infrastructure status to tourism, paving the way for long term State subsidies [18]. MT is thus a beneficiary of this support while the allocations for health have remained below 1.2% of the GDP over the National Five Year Plan periods.

The State itself promoted establishment of corporations of corporate hospitals to manage MT, as in Maharashtra and Gujarat. The Gujarat government announced its Medical Tourism Policy in December 2006, linking its profits with those of the insurance system and professing an annual increase of 33% in MT growth. To promote this ‘business’ it proposed a focus on medical education, manpower development (as of physiotherapists, optometrists, pharmacists and nurses), state of the art diagnostics, blood banks, alternate systems and medi-cities. It set up a high level business council under the Chief Minister to plan the promotion of MT [19]. Thus, MT also became an official reason for supporting medi-cities as in Gurgaon (State of Haryana), Kolkata, and Bangalore and professional attraction towards hi-tech helped the state in this expansion.

### Evidence negating perception of advantages of Mt

The corporate hospital consultants saw increase in GDP, business, employment, professional advancement, competitive spirit, international standard of care with advanced technology, and improved infrastructure as advantages. All of this matched the State’s vision, and the satisfaction of the Indian elite with economic growth rates and its world class medical care is seen as evidence of success. However, debates on the destructive impact of the corporatisation of medicine, the emergence of MT, and social responsibility/irresponsibility of the corporate sector [20] show the other side of reality. But an understanding of the roots of MT and its larger implications evaded the majority of our respondents. Several serious implications have been recognised in the literature:

– i. Direct and indirect shift of subsidies for corporate sector. The former itself is calculated to be Rs.57,000 crores [14].

– ii. MT is overshadowing the poor state of Indian public hospitals as the 11^th^ Plan focused on the former and glamorised the institutions catering to MT [3].

– iii. Rise in prices of medical services is making it even less accessible and more inequitable. The logic of price rise is the need for profit maximisation to break even the initial investment of around Rs.100 crores for a 200 bed corporate hospital. It takes some 4-5 years to break even and some 7-8 years to make reasonable profits. Investment in hospitals is characterised by low returns – the profit rates are around 13% lower than that in IT, finance, or retail [21].

– iv. Rising numbers of establishments of profitable super specialisation and focus on life style surgeries, assisted reproduction, orthopaedic, eyes and cardiac services are distorting priorities as epidemiological reality becomes irrelevant for investors [22].

– v. Overemphasis on tertiary care is leading to shortage of skilled work force in primary care and distorted manpower planning [23].

– vi. Brain drain both external and internal [24].

– vii. Hi-tech institutions act as a push factor for insurance businesses which keep the poor out in India yet, the potential for earning revenues through MT will become an important argument for private hospitals demanding more subsidies from the government in the long run and further undermining services for the poor [25]. Yet there is evidence to show lack of transparency and unethical practices in these hi-tech institutions in India [6].

These facts however, did not constitute the basis of perceptions of even the 27.5 percent of those who were in principle against MT in public sector tertiary hospitals or the 9.6 percent of the private sector physicians who felt it did not help the poor. Both rejected MT on ethical and experiential grounds, the former more openly than the latter.

Despite the fact that patients from the developed world seek MT to save costs in elective treatment (plastic surgery, cosmetic, dental and wellness treatment) or if they are under and uninsured, MT may not be a solution forever. If the efforts of developed nations to restrict losses they incur by cross-border patient travel, by creating their own security systems, succeed, then the profitability of MT may decline. Developed nations are changing insurance laws for those who use MT [10], and developing collective mechanisms of cross border treatment at regulated prices as in the European Union [26] or introducing health Reforms as in the US [27] to handle the crisis of health services. What will then happen to the huge five star high-tech hospitals in India, once their clientele falls? Already many of them survive on heavy State subsidy and by converting hospitals into restaurants, pastry, gift and glossy book shops. Even when it contributes to the growth-oriented economy, its benefits exclude the majority. Is it then an impending disaster, the costs of which will be shifted on to the State and the exchequer as in the case of the Commonwealth Games [28]?

Under these rather alarming circumstances, where India’s health service system is responding to the needs of an elite international and national community, the role of physicians committed to the majority of Indian patients acquires great significance. Their silence and indifference can harm the common patients. Therefore, an over-arching understanding of MT and its implications for the public health system is crucial. It helps one to realise that MT is for cross-border trade in medical care, transfer of technology, manpower, and knowledge that favours the elite and not necessarily the ordinary users of public sector tertiary hospitals. This realisation then provides a more objective basis to their demand for essential policy changes.

### Endnote

^a^All identifying information has been removed and replace with XXXX to ensure anonymity.

## Competing interests

The authors declare that they have no competing interests.

## Authors’ contributions

Both the authors IQ and SR have contributed equally to the study, from conceptualising the problem, review of literature and designing research and formulating the tools. Under the guidance of IQ, SR along with the research team conducted the field work. Both the authors have analysed and written the paper. Both the authors have read and approved the final manuscript.

## Supplementary Material

Additional file 1 Interview schedule for senior physicians in public and corporate hospitals. Click here for file

Additional file 2 Probes. Click here for file
