# Supplementary material for: Medical tourism in india: perceptions of physicians in tertiary care hospitals
Source: Philos Ethics Humanit Med. 2013 Dec 17;8:20. doi: 10.1186/1747-5341-8-20 (PMC3901785; doi:10.1186/1747-5341-8-20)
Supplement: Additional file 1 — Interview schedule for senior physicians in public and corporate hospitals. [file 1747-5341-8-20-S1.doc]

# **INTERVIEW SCHEDULE FOR PUBLIC/ CORPORATE HOSPITAL SENIOR DOCTORS**

1. Name (optional)-
2. Hospital Name- Public/ Corporate
3. Specialization-
4. Whether on regular basis or consultant?
5. How many years of serving in this hospital?
6. Past experience-
7. Roughly how many out door patients visit the OPD of your department every day.
8. How many patients do you see in a day in the OPD?
9. How many indoor patients are admitted daily?
10. What are the main problems of patient care in public hospitals/ corporate hospitals?
11. Are you familiar with the concept of Medical Tourism? (Definition or perception)
12. If you know, what are your views about medical tourism?
13. Do you know that some public hospitals are encouraged to introduce Medical Tourism? Yes/ No
14. What are the strategies used to promote MT?
15. Is Medical Tourism relevant for public sector/ corporate hospitals? If yes, How?
16. If your hospital is listed for Medical Tourism, how will it affect the hospital?
17. What are the implications of MT on general health services?
18. Is private sector, especially corporate sector attracting public sector doctors- yes/ no.
19. If yes, what according to you are the reasons?

20. What are the strengths and weaknesses of working in public/ corporate hospitals apart from monetary benefits?

1. In your knowledge how many doctors shifted from public hospital to corporate?
2. To improve the working of public/ corporate hospitals, what would be your recommendations?

23. What are the government policies to promote medical tourism in both public and corporate sector?
