# Supplementary material for: Medical tourism in india: perceptions of physicians in tertiary care hospitals
Source: Philos Ethics Humanit Med. 2013 Dec 17;8:20. doi: 10.1186/1747-5341-8-20 (PMC3901785; doi:10.1186/1747-5341-8-20)
Supplement: Additional file 2 — Probes. [file 1747-5341-8-20-S2.docx]

**Probes**

**Q10- What are the main problems of patient care in public hospitals/ corporate hospitals?**

Probe 1- List the problems?

Probe 2- What was the exact nature of this problem?

Probe 3- How it affected patient care in your hospital?

**Q 12- What are your views about medical tourism?**

Probe 1- Explain the reasons behind your views?

Probe 2- How benefits accrued or losses impact working of the hospitals.

Probe 3- Impacts on quality of care for general patients.

**Q.14 - What are the strategies used to promote MT?**

Probe 1- How these strategies promote MT?

Probe 2- Who bears the expenses?

Probe 3- Suggest the remaining strategies and probe if these are considered desirable?

**Q.15. Is Medical Tourism relevant for public sector/ corporate hospitals? If yes, How?**

Probe 1- How much revenue is generated?

Probe 2- How is it reinvested?

Probe 3- How it stops brain drain?

Probe 4- How MT makes hospitals efficient?

**Q 16- If Your hospital is listed for Medical Tourism, how will it affect the hospital?**

Probe 1- How are infrastructure, resources and general patient care affected?

Probe 2- Does this affect the care of poor patient coming in corporate/ public hospitals?

Probe 3- Does it reduce or enhance the quality of care for general patients?

**Q.17- What are the implications of MT on general health services?**

Probe 1- What according to you could be the areas of implications?

Probe 2-Other than general patients in the hospital, does MT has implications for health services at any other sites.

Probe 3- Explore all three- Primary Health Care, referral and national priorities.

Probe 4-How do you think it works at these levels?

**Q. 20. What are the strengths and weaknesses of working in public/ corporate hospitals apart from monetary benefits?**

Probe 1- What is the importance of these in your professional work?

Probe 2- Which of these are not desirable?

Probe 3- Where do you think the possibility of these strengths / weaknesses is more; In public or corporate?

**Q. 23 What are the government policies to promote medical tourism in both public and corporate sectors?**

Probe 1- What are the areas of government’s policy intervention?

Probe 2-In which area government is most active?

Probe 3- Where do you think government should act?

Probe 4- Can you suggest improvements in policy?
